# Postprandial enrichment of triacylglycerol-rich lipoproteins with omega-3 fatty acids: lack of an interaction with apolipoprotein E genotype?

**DOI:** 10.1186/1476-511X-13-148

**Published:** 2014-09-16

**Authors:** Valérie Conway, Marie-Julie Allard, Anne-Marie Minihane, Kim G Jackson, Julie A Lovegrove, Mélanie Plourde

**Affiliations:** Centre de recherche sur le vieillissement, Institut Universitaire de Gériatrie de Sherbrooke, Université de Sherbrooke, 1036 Belvédère Sud, Sherbrooke, J1H 4C4 Canada; Hugh Sinclair Unit of Human Nutrition and Institute for Cardiovascular and Metabolic Research (ICMR), University of Reading, Reading, UK; Nutrition Department, Norwich Medical School, University of East Anglia, Norwich, UK

**Keywords:** *APOE4* carriers, Chylomicrons, *n −* 3 PUFA metabolism, VLDL, Fish-oil supplementation, Postprandial lipemia

## Abstract

**Background:**

We have previously demonstrated that carrying the apolipoprotein (apo) E epsilon 4 (*E4+*) genotype disrupts omega-3 fatty acids (*n −* 3 PUFA) metabolism. Here we hypothesise that the postprandial clearance of *n −* 3 PUFA from the circulation is faster in *E4+* compared to non-carriers (*E4−*). The objective of the study was to investigate the fasted and postprandial fatty acid (FA) profile of triacylglycerol-rich lipoprotein (TRL) fractions: S_f_ >400 (predominately chylomicron CM), S_f_ 60 − 400 (VLDL_1_), and S_f_ 20 − 60 (VLDL_2_) according to *APOE* genotype.

**Methods:**

Postprandial TRL fractions were obtained in 11 *E4+* (ϵ3/ϵ4) and 12 *E4−* (ϵ3/ϵ3) male from the SATgenϵ study following high saturated fat diet + 3.45 g/d of docosahexaenoic acid (DHA) for 8-wk. Blood samples were taken at fasting and 5-h after consuming a test-meal representative of the dietary intervention. FA were characterized by gas chromatography.

**Results:**

At fasting, there was a 2-fold higher ratio of eicosapentaenoic acid (EPA) to arachidonic acid (*P* = 0.046) as well as a trend towards higher relative% of EPA (*P* = 0.063) in the S_f_ >400 fraction of *E4+*. Total *n −* 3 PUFA in the S_f_ 60 − 400 and S_f_ 20 − 60 fractions were not *APOE* genotype dependant. At 5 h, there was a trend towards a time × genotype interaction (*P* = 0.081) for EPA in the S_f_ >400 fraction. When sub-groups were form based on the level of EPA at baseline within the S_f_ >400 fraction, postprandial EPA (%) was significantly reduced only in the high-EPA group. EPA at baseline significantly predicted the postprandial response in EPA only in *E4*+ subjects (*R*^*2*^ = 0.816).

**Conclusion:**

Despite the DHA supplement contain very low levels of EPA, *E4+* subjects with high EPA at fasting potentially have disrupted postprandial *n −* 3 PUFA metabolism after receiving a high-dose of DHA.

**Trial registration:**

Registered at clinicaltrials.gov/show/NCT01544855.

## Background

A higher intake of fish-oil containing long chain *n* − 3 polyunsaturated fatty acids (*n* − 3 PUFA) is known to decrease the risks of cardiovascular disease (CVD) [1] and potentially cognitive decline in the elderly [2]. However, carriers of the apolipoprotein (apo) E ϵ4 allele (*E4*+), the most important genetic risk factor for Alzheimer’s disease (AD) [3], do not seem to be protected against cognitive decline through fish consumption [4]. In humans, three principal isoforms of *APOE* (i.e. *APOE2*, *APOE3* and *APOE4*) resulting from six genotypes (i.e. ϵ2/ϵ2, ϵ2/ϵ3, ϵ2/ϵ4, ϵ3/ϵ3, ϵ3/ϵ4 and ϵ4/ϵ4) have been identified [5]. Even if results are still inconsistants, most studies aiming to investigate the response to fish-oil supplementation according to *APOE* genotype suggested greater lipid responsiveness in *E4*+ carriers compared to non-carriers (*E4*−).

ApoE is found on the surface of chylomicron remnants, VLDL and HDL_2_ particles where it acts as a ligand for low density lipoprotein receptor family (LDLR), thus playing an important role in the regulation and transport of exogenous (i.e. dietary lipids) and endogenous (i.e. derived from the liver) fatty acids (FA) [6]. After a high-fat meal, plasma triacylglycerol (TG) levels are temporarily raised through hepatic (i.e. from VLDL) as well as intestinally derived lipids (i.e. from chylomicron particles) [7]. This normal transitory rise in blood TG is referred to as postprandial lipemia. In literature, *APOE* genotype has been proposed as an important genetic determinant of the interindividual variability in postprandial lipemia [8, 9]. The differencial affinity of *APOE* isoform for its clearence receptors, the preferential incorporation of apoE4 within TG-rich lipoproteins (TRL), and its modulating action on size and lipid composition of plasma TRL may all explain part of the *APOE* genotype-induce variability in lipid metabolism [6, 10].

Recently, Liang *et al.* [11] reported that *APOE* genotype was not significantly associated to differencial modification in levels of DHA and EPA within TC, TG or LDL-C, but was significantly associated to their concentrations with HDL-C. Supporting differential lipid metabolism between *E4*+ and *E4*− subjects, our previous results have demonstrated that postprandial *β*-oxidation of DHA was higher in *E4*+ and that increment of plasma level of DHA following fish-oil supplementation was reduced in this population [12, 13]. Studying the metabolism of *n* − 3 PUFA in *E4*+, by understanding how *n* − 3 PUFA are transported in TRL, could help establish effective *n* − 3 PUFA dosage and duration for disease prevention in *E4*+ patient.

Our hypothesis is that the clearance of *n* − 3 PUFA is faster in *E4*+ subjects, resulting in lower% of circulating *n* − 3 PUFA in postprandial TRL after acute DHA intake. This study is a secondary analysis looking at the responses to an 8-wk diet high in fat and saturated fat supplemented with 3.45 g/d of DHA (HSF-DHA diet) and to the postprandial response to a macronutrient matching HSF-DHA test-meal according to *APOE* genotype. Therefore, our objective was to determine the FA profile in different TRL (Svedberg flotation rate: S_f_ >400 (predominately chylomicrons), S_f_ 60 − 400 (very low density lipoprotein 1; VLDL_1_) and S_f_ 20 − 60 (VLDL_2_)) isolated at fasting (0 h) and 5-h after eating a high-fat test-meal containing DHA (3.45 g) in *E4+* and *E4−* men.

## Results

### Participants

The characteristics of the *E4*− and *E4*+ participants matched for age and BMI are shown in Table 1. There was no difference in plasma lipids or glucose levels between the two genotype groups following the 8-wk HSF-DHA diet − referred as baseline. The plasma concentration of apoE differs significantly between *E4*− and *E4*+ participants since *E4*+ had 28% less plasma apoE (μg/mL) when compared to non-carriers after the 8-wk HSF-DHA diet. The test-meal was well tolerated by all participants. Only one of the recruited subjects (from the *E4+* group) dropped the study protocol.

**Table 1:** **Baseline anthropometric and biochemical data for carriers (** ***E4*** **+) and non-carriers (** ***E4*** **−) of an** ***APOE4*** **allele following the 8-wk HSF-DHA diet**

Anthropometric	Whole group (N = 23)	***E4***− (N = 12)	***E4***+ (N = 11)	***P***genotype
Age (y)	54 ± 2	52 ± 2	56 ± 3	0.346
BMI (kg/m^2^)	25.97 ± 0.57	26.53 ± 0.72	25.36 ± 0.91	0.319
**Biochemical**				
Total cholesterol (mmol/L)	5.67 ± 0.13	5.77 ± 0.22	5.57 ± 0.16	0.478
HDL-C (mmol/L)	1.45 ± 0.06	1.48 ± 0.08	1.42 ± 0.11	0.649
LDL-C (mmol/L)	4.04 ± 0.16	4.13 ± 0.26	3.96 ± 0.20	0.615
TG (mmol/L)	1.07 ± 0.09	1.03 ± 0.12	1.11 ± 0.15	0.681
NEFA (μmol/L)	378.1 ± 27.3	379.1 ± 33.6	377.0 ± 45.6	0.970
ApoE (μg/mL)	37.33 ± 2.27	41.69 ± 3.51	32.58 ± 2.14	**0.042**
Glucose (mmol/L)	5.68 ± 0.08	5.58 ± 0.10	5.78 ± 0.11	0.205
CRP (μg/mL)	1.28 ± 0.31	1.07 ± 0.29	1.50 ± 0.57	0.501

Values are presented as mean ± SEM. *P* values were obtained using one-way ANOVA in SPSS version 22.0 (IBM Corp., Armonk, NY). Bold characters are used to indicate significant effects (*P* < 0.05).

*ApoE*: Apolipoprotein E; *BMI*: Body mass index; *CRP*: C-reactive protein; *HDL-C*: High-density lipoprotein-cholesterol; *LDL-C*: Low-density lipoprotein-cholesterol; *NEFA*: Non-esterified fatty acids; *TG*: Triacylglycerol.

### Fatty acid profile of TRL

**Figure 1:**
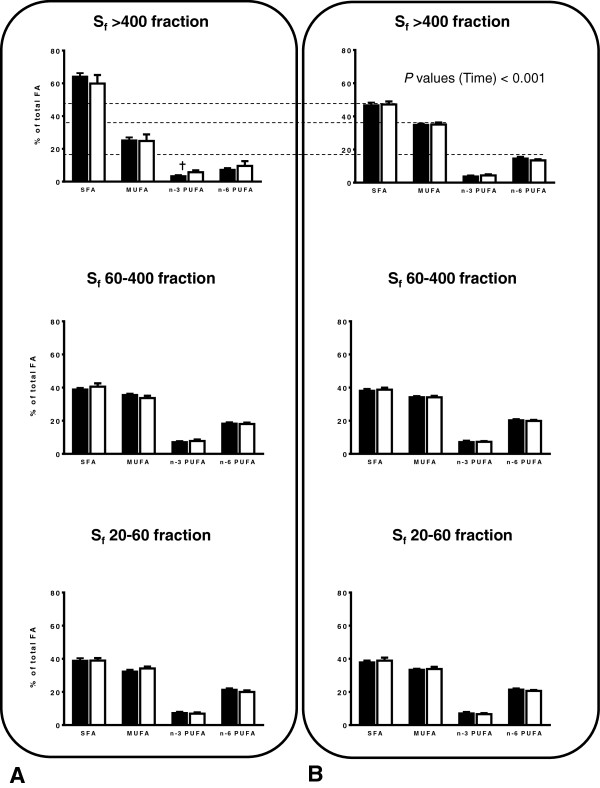
**Fatty acid profile of TRL (top figures, S**_**f**_ **>400; middle figures, S**_**f**_ **400 − 60; bottom figures, S**_**f**_ **20 − 60) in** ***E4−*** **(**■**) and** ***E4+*** **(**□**) at A) fasting and B) in postprandial.** ANOVA was used to investigate the presence of significant difference according to time and according to *APOE* genotype for the relative% of saturated fatty acids (SFA), monounsaturated fatty acids (MUFA), omega-3 and omega-6 fatty acids (*n* − 3 PUFA and *n* − 6 PUFA). ^✝^ Trend effect for genotype (*P* = 0.084).

The fatty acid profile was performed after the participants received the HSF-DHA diet for 8 weeks. Overall, there was limited *APOE* genotype effect on fasting and postprandial FA profile of the TRL fractions (Figure 1A and B).

#### S_f_ >400 fraction

Following the 8-wk DHA enriched diet, the relative% of fasting DHA in S_f_ >400 fraction significantly predicted the level of plasma TG, independently of *APOE* genotype (*R*^*2*^ = 0.468, *P* = 0.010, data not shown). Besides a trend towards higher relative% of EPA and *n*‒3 PUFA at fasting in *E4*+ subjects compared to *E4*− (*P* = 0.084, Figure 1A), all other FA did not differ according to *APOE* genotype (Table 2). The ratio of EPA to arachidonic acid (AA, 20:4 *n* − 6) was >2-fold higher in S_f_ >400 fraction of *E4*+ subjects compared *E4*− with respective values of 2.4 ± 0.8 vs. 1.1 ± 0.5 (*P* = 0.046, data not shown). At fasting, 16:0 (34%), 18:0 (24 − 27%) and 18:1 *n* − 9 (22%) were the main FA whereas alpha-linoleic acid (ALA, 18:3 *n* − 3) composed 47% of total *n* − 3 PUFA (Table 2). There was a significant correlation between the relative% of DHA at fasting and the concentration of triacylglycerol (TG), non-esterified FA (NEFA), small and dense LDL-C and C-reactive protein (CRP) concentrations within the S_f_ >400 fraction. Similarly, the relative% of 18:2 *n* − 6 at fasting was correlated to the concentration of small and dense LDL-C (*r* = 0.615, *P* = 0.025, data not shown).

**Table 2:** **Fatty acid profiles of S**_**f**_ **>400 fractions in response to acute docosahexaenoic acid (DHA) intake in carriers (** ***E4*** **+) and non-carriers (** ***E4*** **−) of an** ***APOE4*** **allele**

		Fasting	Postprandial	Change	***P***values		
		(relative%)	(relative%)	(Δ)	Interaction	Genotype	Time
16:0	*E4+*	33.44 ± 2.05	33.75 ± 0.77	+ 0.31	0.457	0.833	0.487
	*E4−*	34.26 ± 1.49	33.76 ± 1.01	+ 0.50			
18:0	*E4+*	23.75 ± 2.92	10.61 ± 1.65	− 13.14	0.482	0.310	**<0.001**
	*E4−*	27.12 ± 1.73	9.74 ± 1.03	− 17.38			
16:1 *n* − 7	*E4+*	1.44 ± 0.18	0.44 ± 0.08	− 1.00	0.467	0.174	**0.011**
	*E4−*	1.46 ± 0.13	0.74 ± 0.20	− 0.72			
18:1 *n* − 9	*E4+*	21.74 ± 4.21	33.70 ± 1.18	+ 11.96	0.708	0.969	**<0.001**
	*E4−*	22.51 ± 1.54	33.12 ± 0.72	− 10.61			
18:2 *n* − 6	*E4+*	8.63 ± 2.90	12.51 ± 0.68	+ 3.88	0.219	0.520	**0.001**
	*E4−*	5.69 ± 0.88	13.31 ± 1.05	+ 7.62			
20:4 *n* − 6	*E4+*	0.78 ± 0.12	0.77 ± 0.08	− 0.01	0.547	0.265	0.757
	*E4−*	1.44 ± 0.65	0.98 ± 0.11	− 0.46			
18:3 *n* − 3	*E4+*	2.68 ± 0.85	0.89 ± 0.08	− 1.79	0.196	0.309	**0.015**
	*E4−*	1.60 ± 0.37	0.93 ± 0.07	− 0.67			
20:5 *n* − 3	*E4+*	1.52 ± 0.29	0.86 ± 0.10	− 0.66	0.081	0.251	0.154
	*E4−*	0.90 ± 0.21	0.86 ± 0.13	− 0.04			
22:6 *n* − 3	*E4+*	1.50 ± 0.65	2.53 ± 0.48	+ 1.03	0.949	0.219	**0.024**
	*E4−*	0.75 ± 0.16	1.96 ± 0.45	+ 1.21			

Values are presented as mean relative percentages ± SEM or as change (Δ) compared to fasted state (i.e.% at postprandial value –% at fasting value). *P* values were obtained using a Factorial Repeated Measures (Split-Plot) ANOVA in SPSS version 22.0 (IBM Corp., Armonk, NY). Bold characters are used to indicate significant effects (*P* < 0.05).

Five hours after receiving the test meal, none of the tested FA in the S_f_ >400 fraction were *APOE* genotype-dependant (Table 2). There was no significant time × genotype effect for any of the FA (Table 2). However, there was a trend towards a significant time × genotype interaction on the relative% of EPA (*P* = 0.081). The relative% of EPA in postprandial was strongly correlated with DHA (*r* = 0.875, *P* < 0.001, data not shown). Five hours after receiving the HSF-DHA test-meal, the postprandial relative% of SFA in the S_f_ >400 was significantly reduced compared to fasting, whereas the relative% of both MUFA and total *n* − 6 PUFA were increased. These time effects were independent of *APOE* genotype (Figure 1B).

**Figure 2:**
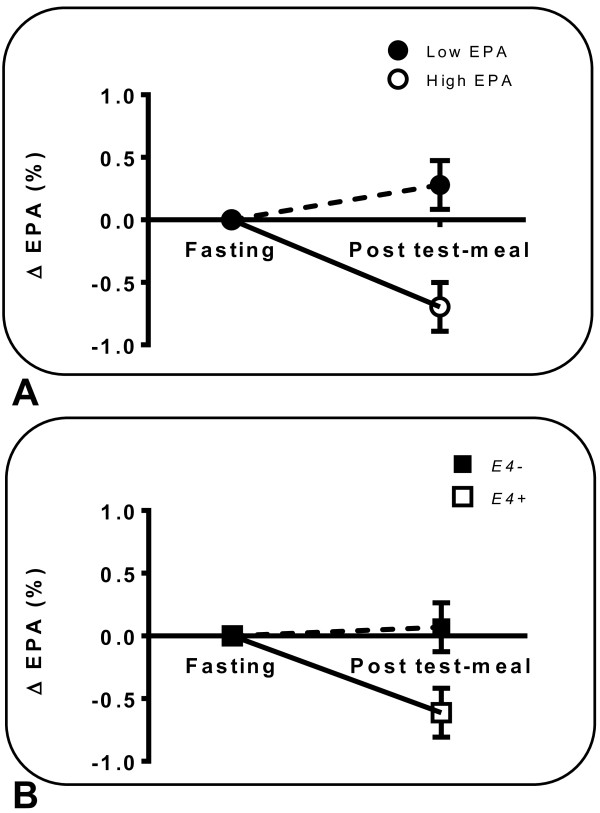
**Changes in the eicosapentaenoic acid (ΔEPA) content of postprandial S**_**f**_ **>400 lipoproteins depending on: A) EPA-status at fasting (N = 11 or 12/group); B)** ***APOE*** **genotype (N = 11 or 12/group)** ***; E4−*** **(**■**) and** ***E4+*** **(**□**).** Median fasting level of EPA in S_f_ >400 lipoproteins was used to create the low <1.0% (●) and high >1.0% sub-groups (○). Factorial Repeated Measures (Split-Plot) ANOVA was used to investigate the presence of significant interaction. *P* value for EPA-status at fasting × genotype interaction was = 0.036.

EPA level at fasting, had a significant impact on the postprandial response (ΔEPA) to the test-meal (*P* = 0.021) Since EPA level at fasting tended to change the postprandial response of EPA within the S_f_ >400 fraction, two groups were formed based on the median level of EPA at fasting in the S_f_ >400 fraction (low-EPA sub-group = EPA < 1.0% vs. high-EPA sub-group = EPA > 1.0%). There was a significant interaction between fasting EPA-status (i.e. low or high) and *APOE* genotype (*P* interaction = 0.036; Figure 2). Postprandial EPA was 7-fold lower in participants in the high-EPA sub-group than in the low-EPA sub-group (Figure 2A). Similarly*,* the postprandial response for EPA (ΔEPA) after the test-meal was greater in *E4*+ subjects (Figure 2B). In multiple regression analysis, the relative% of EPA in S_f_ >400 lipoproteins at fasting, significantly predicted the postprandial response in EPA (ΔEPA) only in *E4*+ subjects (*R*^*2*^ = 0.816, *P* < 0.014, data not shown).

#### S_f_ >60 − 400 fraction

At fasting, relative% of all tested FA were independent of *APOE* isoforms. At fasting, the fatty acid profile of the S_f_ 60 − 400 fraction was mainly 16:0 (29%), SA (8 − 9%) and 18:1 *n*‒9 (30%). The main *n* − 3 PUFA was DHA (Table 3).

**Table 3:** **Fatty acid profiles of S**_**f**_ **60 − 400 fractions in response to acute docosahexaenoic acid intake in carriers (** ***E4*** **+) and non-carriers (** ***E4*** **−) of an** ***APOE4*** **allele**

		Fasting	Postprandial	Change	***P***values		
		(relative%)	(relative%)	(Δ)	***Interaction***	***Genotype***	***Time***
16:0	*E4+*	29.01 ± 1.35	30.21 ± 0.74	+ 1.20	0.981	0.688	0.063^†^
	*E4−*	28.54 ± 0.73	29.77 ± 0.73	+ 1.23			
18:0	*E4+*	9.15 ± 1.27	5.96 ± 0.65	− 3.19	0.452	0.534	**<0.001**
	*E4−*	8.07 ± 0.71	5.80 ± 0.47	− 2.27			
16:1 *n* − 7	*E4+*	3.12 ± 0.39	2.34 ± 0.36	− 0.78	0.676	0.823	**<0.001**
	*E4−*	3.27 ± 0.27	2.38 ± 0.24	− 0.89			
18:1 *n* − 9	*E4+*	28.93 ± 0.12	30.29 ± 0.81	+ 1.36	0.307	0.326	0.415
	*E4−*	30.52 ± 0.52	30.36 ± 0.42	− 0.16			
18:2 *n* − 6	*E4+*	15.84 ± 0.66	17.41 ± 0.48	+ 1.57	0.883	0.912	**0.001**
	*E4−*	15.97 ± 0.55	17.42 ± 0.55	+ 1.45			
20:4 *n* − 6	*E4+*	1.91 ± 0.17	2.05 ± 0.14	+ 0.14	0.648	0.449	**0.047**
	*E4−*	2.03 ± 0.16	2.25 ± 0.16	+ 0.22			
18:3 *n* − 3	*E4+*	1.64 ± 0.19	1.35 ± 0.12	− 0.29	0.791	0.409	**0.018**
	*E4−*	1.49 ± 0.09	1.25 ± 0.10	− 0.24			
20:5 *n* − 3	*E4+*	1.19 ± 0.13	1.25 ± 0.04	− 0.06	0.526	0.948	0.154
	*E4−*	1.32 ± 0.30	1.15 ± 0.11	− 0.17			
22:6 *n* − 3	*E4+*	3.74 ± 0.43	4.33 ± 0.33	+ 0.59	0.878	0.497	0.099
	*E4−*	3.44 ± 0.45	3.91 ± 0.47	+ 0.47			

Values are presented as mean relative percentages ± SEM or as change (Δ) compared to fasted state (i.e.% at postprandial value –% at fasting value). *P* values were obtained using a Factorial Repeated Measures (Split-Plot) ANOVA in SPSS version 22.0 (IBM Corp., Armonk, NY). Bold characters are used to indicate significant effects (*P* < 0.05). ^†^Trend effect was set at *P* < 0.09.

Five hours after the HSF-DHA test-meal, the relative% of all tested FA were independent of the *APOE* genotype (Table 3). DHA represented 59% and 62% of total *n* − 3 PUFA in *E4*− and in *E4*+ respectively. There was no significant time × genotype interaction or independent genotype effect on the postprandial FA profile of S_f_ 60 − 400 lipoproteins (Table 3). The relative% of 18:2 *n‒*6 in postprandial was positively correlated to LDL-C and the relative% of 18:3 *n*‒3 was positively correlated to HDL-C (data not shown).

#### S_f_ 20 − 60 fraction

At fasting, relative% of all tested FA were independent of *APOE* isoforms with the exception of, which was significantly lower in *E4*+ compared to *E4*− (Table 4). At fasting, 16:0 (28%), 18:0 (9%) and 18:1 *n*‒9 (28 − 30%) were the main FA. Half of total *n* − 3 PUFA was DHA. The relative% of AA at fasting was significantly correlated to plasma concentration of apoE (*r* = 0.496, *P* = 0.019; data not shown). In multiple regression, HDL-C concentration was able to predict the variation of AA at fasting (%) only in *E4*+ subjects (*R*^*2*^ = 0.492, *P* = 0.010; data not shown).

**Table 4:** **Fatty acid profiles of S**_**f**_ **20 − 60 fractions in response to acute docosahexaenoic acid intake in carriers (** ***E4*** **+) and non-carriers (** ***E4*** **−) of an** ***APOE4*** **allele**

		Fasting	Postprandial	Change	***P***values		
		(relative%)	(relative%)	(Δ,%)	***Interaction***	***Genotype***	***Time***
16:0	*E4+*	28.40 ± 1.07	29.71 ± 1.24	+ 1.31	0.535	0.330	0.193
	*E4−*	27.69 ± 0.75	28.61 ± 0.80	+ 0.92			
18:0	*E4+*	8.72 ± 0.53	7.12 ± 0.86	− 1.60	0.775	0.619	**0.001**
	*E4−*	9.29 ± 0.67	7.28 ± 0.46	− 2.01			
16:1 *n* − 7	*E4+*	2.49 ± 0.27	2.25 ± 0.26	− 0.24	0.630	0.921	**<0.001**
	*E4−*	2.60 ± 0.20	2.26 ± 0.18	− 0.34			
18:1 *n* − 9	*E4+*	29.79 ± 0.94	29.83 ± 1.17	+ 0.04	0.166	0.381	0.148
	*E4−*	28.01 ± 0.92	29.45 ± 0.43	+ 1.44			
18:2 *n* − 6	*E4+*	17.12 ± 0.85	17.69 ± 0.47	+ 0.57	0.552	0.259	0.164
	*E4−*	17.84 ± 0.55	17.96 ± 0.49	+ 0.12			
20:4 *n* − 6	*E4+*	2.22 ± 0.16	2.39 ± 0.18	+ 0.17	0.488	**0.022**	0.681
	*E4−*	2.91 ± 0.23	2.77 ± 0.19	− 0.14			
18:3 *n* − 3	*E4+*	1.31 ± 0.16	1.19 ± 0.09	− 0.12	0.367	0.755	**0.025**
	*E4−*	1.35 ± 0.12	1.07 ± 0.05	− 0.28			
20:5 *n* − 3	*E4+*	1.42 ± 0.17	1.24 ± 0.11	− 0.18	0.746	0.991	0.133
	*E4−*	1.39 ± 0.16	1.25 ± 0.11	− 1.14			
22:6 *n* − 3	*E4+*	3.74 ± 0.53	3.77 ± 0.38	+ 0.03	0.779	0.706	0.896
	*E4−*	4.08 ± 0.46	3.82 ± 0.52	− 0.26			

Values are presented as mean relative percentages ± SEM or as change (Δ) compared to fasted state (i.e.% at postprandial value –% at fasting value). *P* values were obtained using a Factorial Repeated Measures (Split-Plot) ANOVA in SPSS version 22.0 (IBM Corp., Armonk, NY). Bold characters are used to indicate significant effects (*P* < 0.05).

Five hours after the HSF-DHA test-meal, the relative% of FA were all independent of the *APOE* genotype (Table 4). There was no significant time × genotype interaction for any of the tested FA (Table 4). The relative% of 18:3 *n*‒3 at fasting was positively correlated to the postprandial level of ALA (*r* = 0.553, *P* = 0.009), EPA (*r* = 0.611, *P* = 0.003), DHA (*r* = 0.479, *P* = 0.028) and to plasma concentration of HDL-C (*r* = 0.592, *P* = 0.004). It was also negatively correlated to plasma concentration of TG (*r* = −0.510, *P* = 0.015) (data not shown).

## Discussion

Contrary to our hypothesis, total *n* − 3 PUFA levels of the TRL fractions after a chronic DHA supplementation for 8-wk (3.45 g/d) was not significantly dependent on *APOE* genotype. However, there was a >2-fold higher ratio of EPA:AA in the S_f_ >400 fraction as well as a trend towards higher levels of EPA at fasting in *E4+* subjects compared to *E4‒*. When groups were formed based on fasting EPA in S_f_ >400 lipoproteins, the postprandial response for EPA was greater in *E4+* from the high-EPA sub-group. EPA enrichment of lipoproteins at fasting predicted more than 80% of the EPA-response in *E4+* subjects following the prescribed DHA supplementation for 8-wk (3.45 g/d).These results suggests that in *E4+,* the response to fish-oil supplementation is dependant of the fasting EPA-status.

The greater reduction of EPA in the S_f_ >400 lipoproteins of the *E4 +* from the high fasting EPA sub-group, may potentially be explained by enhanced hepatic uptake of large TRL. In accordance with this hypothesis, apoE4 has a greater affinity for the low density lipoprotein receptor (i.e. LDL-R), hence, enhancing its clearance from blood. Since apoE4 preferentially incorporates into large TLR particles [6, 10], Sf >400 lipoproteins may be preferentially and more rapidly cleared in *E4 +*.Therefore, EPA clearance may be faster in *E4 +* and EPA may thereafter be preferentially catabolized or metabolized in the liver due to its poor incorporation into cell membranes . Thereafter, subjects may preferentially catabolize EPA. This hypothesis is supported by results from *in vivo* and *in vitro* experiments in which TG-lowering action of fish-oil supplementation was explained throughout enhance mitochondrial lipid catabolism (i.e. *β*-oxidation) [14, 15]. As explained by Olano-Martin *et al.* [16], reduction of TG after EPA and/or DHA supplementation is partially attributable to up-regulation of genes involved in FA catabolism. Recent work from our group suggested that FA uptake and FA *β*-oxidation by the liver of apoE-targeted replacement mice expressing human *APOE4* genotype is enhance through increase concentrations of hepatic carnitine acyl­transferase (CPT1), the limiting enzyme of FA *β*-oxidation [17]. Moreover, FA preference towards *β*-oxidation may be modify in *E4*+ as suggested in a previous study [13]. The greater reduction of EPA, with no apparent reduction in DHA, can be related to preferential catabolism of EPA over DHA, as DHA is a much poorer substrate than EPA for the acyl-CoA synthase [14, 18]. These latter hypotheses and the underling mechanisms are as yet speculative and need further investigation. Even if our results seem to suggest that the isoform-specific action of *APOE* genotype is limited and mostly affects FA profile of large TRL (i.e. S_f_ > 400 fraction), a complete picture of the *APOE* isoform-specific modulation of *n* − 3 PUFA transport and clearance by adding FA profiles of LDL and HDL particles would have added to the present results − these lipoproteins were not available for us to analyse. However, in accordance with our results, the greater sensitivity of fasting [19] and postprandial [20] TG to dietary DHA intake in *E4+* is usually explained throughout isoform-specific modulation of TLR metabolism rather than to LDL and HDL particles.

Trials investigating DHA supplementation generally report a dose‒dependent increase in both plasma DHA and EPA [21]. For example, in the 8-wk HSF-DHA diet of the SATgenϵ study, there was a 2-fold increase of DHA and EPA in plasma phospholipids in *E4+* and *E4‒* [22]. The lack of *APOE* isoform-specific postprandial response during acute DHA ingestion in this follow-up study could simply result from the enrichment of TRL at baseline, which occurs whilst the HSF-DHA diet for 8-wk [22]. This enrichment may have masked any subtle changes in the postprandial response between *E4*− and *E4*+ subjects. Greater modulation of postprandial response to *n* − 3 PUFA supplementation may be possible through intake of EPA enriched test-meal rather than DHA enriched test-meal. Indeed, Cazzola *et al.* [23] proposed based on their dose–response investigation on normotriacylglycerolaemic males supplemented 1.4 to 4.05 g EPA/day for 12-wk, that EPA may be responsible for the TG-lowering action of fish oil. These hypotheses are speculative and deserve to be tested in future investigation according to *APOE* genotype.

AA was lower in Sf 20 − 60 lipoproteins of *E4+* at fasting. This is an intriguing result since usually AA rarely varies in plasma lipids. A significant reduction in AA (‒14 μmol/L) was reported while studying postprandial response to a 7-week supplementation with *n* − 3 PUFA (1 g/d EPA and DHA) in normolipaemic individuals, with no regards to *APOE* genotype [24]. Thus, the lower level of plasma AA in fasted *E4+* subjects may have result from the greater responsiveness of *E4+* [20] to the prior 8-wk HSF-DHA diet [22]. AA kinetics has never been studied in humans so whether *APOE* genotype significantly changes its kinetics remains to be established.

This study has strengths and limitations. The main limitations are related to the high saturated fat content of the HSF-DHA-rich test meal (53 g of fat), to lipoprotein FA profile in relative% rather than in concentration, and finally to the use of a single postprandial time-point (5 h) for this analysis. Indeed, fat quality and quantity [8], as well as the vehicle in which DHA is administered [25], may all affect postprandial lipid response to acute ingestion and thus, may mask subtle changes according to *APOE* genotype. Finally, providing FA profile in concentrations rather than in relative% would have been informative but unfortunately this analysis was not performed and due to the limited volume of sample available, this analysis cannot be performed. This would have given complementary information regarding FA profiles, especially with regards to the level of each TRL according to *APOE* genotype. This study represents a secondary analysis of a trial designed to investigate dietary fat quality and quantity on postprandial lipid metabolism according to *APOE* genotype [22]. Since recent evidences from another group suggest different metabolic activities after EPA and DHA supplementation [11], and considering that Cazzola *et al.* [23] proposed that EPA may be more potent at reducing plasma TG than DHA, studying the response to chronic and acute EPA intake, on the FA profile of TRL according to *APOE* genotype, and not in a context of HSF diet, would be of great interest.

## Conclusions

This study reported that FA profile of large TRL (S_f_ >400) is the most modified by chronic and acute DHA intake and that it is not *APOE*-isoform dependant. Despite the prior HSF-DHA diet contained very low levels of EPA, *E4+* subjects with high fasting plasma levels of EPA potentially have disrupted postprandial *n −* 3 PUFA metabolism after receiving an acute high-dose of DHA. In future trial design, looking at the interaction between participant’s EPA-status at baseline and *APOE* genotype on the response to EPA and/or DHA supplementation should be considered.

## Methods

### Participants

Twenty-three men − 12 *E4*− (ϵ3/ϵ3) and 11 *E4+* (ϵ3/ϵ4) − participating in the SATgenϵ study also undertook postprandial studies at the end of three iso-energetic 8-wk diets: 1) low-fat diet (LF); 2) high-fat, high saturated fat (HSF); 3) HSF + 3.45 g/day DHA (HSF-DHA) diets. All participants consumed the three diets in the same order, without in between washout period. The present postprandial study only investigated the chronic and acute response to HSF-DHA diet in *E4−* and *E4 +* men. Complete details of the SATgenϵ dietary intervention and of the postprandial investigation have been presented in detail elsewhere [20, 22]. Briefly, twenty-four hours prior postprandial intervention, participants were asked to abstain from alcohol and to consume a low-fat meal (<0 g fat) before 8 pm in the evening. The HSF-DHA test-meal (5.4 MJ) used in this postprandial investigation matched the macronutrient composition of the dietary intervention (i.e. HSF-DHA diet) as detailed in [20]. Briefly HSF-DHA meal was composed of (45.1% carbohydrate, 17.0% protein, 37.8% fat, of which 18.3% saturated fatty acids, 12.2% monounsaturated fatty acids, 6.6% polyunsaturated fatty acids with 0.2% EPA and 1.4% DHA). The HSF-DHA test-meal was a warm chocolate drink containing the fish-oil (DHA), with toast and jam. It was provided and consumed within 20-min. The DHA enriched test-meal contained 5.7% saturated fatty acids, 11.6% monounsaturated fatty acids, 82% polyunsaturated fatty acids, 8.3% EPA, 3.1% DPA, and 57.5% DHA, which provided 3.45 g of DHA/d + 0.19 g EPA/d (Croda Healthcare, UK). This study was approved by the University of Reading Research Ethics Committee and is registered at clinicaltrials.gov as NCT01384032. The study was conducted according to the Declaration of Helsinki’s guidelines. Participants of the SATgenϵ trial provided their informed consent for the study.

### Biochemical assessments

After an overnight fast, subjects attended the investigation unit where an indwelling cannula was inserted into a forearm vein. Two blood samples were taken at baseline (i.e. fasting state) and 5-h after consuming the HSF-DHA test-meal (i.e. providing 3.45 g of DHA). Blood samples were transferred to heparin containing tubes and were separated by centrifugation at 1700 × *g* for 10 min in a bench-top centrifuge at 4°C, and stored at −20°C until analysis. In order to prevent the proteolytic degradation of the apolipoproteins, 6 μL EDTA (0.5 mol/L), 3 μL PMSF (10 mmol/L in isopropanol) and 15 μL aprotinin (10 000 kallikrein inactivator units/mL) were added immediately to the plasma. The plasma was stored overnight at 4°C until isolation of the S_f_*>*400 (predominantly chylomicron), S_f_ 60–400 (VLDL_1_) and S_f_ 20–60 (VLDL_2_) fractions using density gradient ultracentrifugation, as previously described [23]. After isolation of TRL fractions (1 mL), recovered volumes were divided into portions and stored at −20°C until further analysed.

### Fatty acid profile analyses

Total lipids were extracted from the TRL fractions (150 μL) using the Folch method [26]. The plasma total lipid extract was then saponified using 1 M KOH/methanol and heated at 90°C for 1-h, which hydrolyses the fatty acids from cholesterol and glycerol. After cooling to room temperature, hexane and saline were added. The hexane and cholesterol phase were discarded and the remaining saline and fatty acid salt mixture was acidified with HCl to obtain the free fatty acids. Transmethylation of the resulting non-esterified FA into FA methyl esters was performed using boron 14% trifluoride/methanol (Sigma-Aldrich, Saint-Louis, MO). FA methyl esters were analyzed using a gas chromatograph (model 6890, Agilent, Palo Alto, CA) equipped with a 50 m BPX-70 fused capillary column (SGE, Melbourne, Australia) as described in [27].

### Statistics

This study represents a secondary analysis of the SATgenϵ trial conducted at the University of Reading (UK) [20]. Sample size estimation was originally performed on the expected change in postprandial TG-response between genotype groups (i.e. *E4*− vs. *E4*+). The sample size estimation was performed to allow the detection of a minimum of 238 mmol/L × min change in TG-response between genotype groups. A SD of 189 mmol/L × min in response to dietary modifications has been used for sample size calculations. Based on previous studies, the required number per genotype groups was N = 10 (alpha = 0.05 two-tailed, power = 80%) [19]. The same number of subjects (N = 10/group) was required to detect a minimum of 90 nmol/mL × h with a SD of 70 nmol/mL × h change in [^13^C]-DHA metabolism between *E4+* and *E4−* [13] with an anticipated dropout of 15%.

All data were analysed for statistical differences using a Factorial Repeated Measures (Split-Plot) ANOVA in SPSS version 22.0 (IBM Corp., Armonk, NY). Normal distribution and homogeneity of variance were evaluated before further analysis. The main effect of *APOE* genotype on fasting and on the changes (Δ) in serum *n* − 3 PUFA profile was also investigated using non-parametric analysis of variance in SPSS. The median value for EPA relative% in the TRL fraction at baseline was used to create groups (i.e. low-EPA group or high-EPA group). The impact of EPA-status at baseline (i.e. Low or High) and *APOE* genotype on the response (Δ) to HSF-DHA test-meal was also investigated using factorial (two-way) ANOVA analysis of variance in SPSS. When interaction terms were found significant, the interaction was presented graphically. Bivariate Pearson correlation analysis and multivariate stepwise regression models were used to investigate associations among outcomes. Data are presented as means ± SEMs and *P* values <0.05 were considered statistically significant. Trend effect was set at *P* values ≤ 0.085. Each FA is presented in relative% compared to total-FA identified or as postprandial response (Δ):


## Abbreviations

AA: Arachidonic acid
ALA: α-linoleic acid
Apo: Apolipoprotein
ApoE: Apolipoprotein E
CRP: C-reactive protein
CVD: Cardiovascular disease
DHA: Docosahexaenoic acid
*E4+*: *APOE* epsilon 4 carriers
*E4−*: *APOE* epsilon 4 non-carriers
EPA: Eicosapentaenoic acid
FA: Fatty acid
HDL: High density lipoprotein
HSF: High saturated fat
LDL: Low density lipoprotein
MUFA: Monounsaturated fatty acids
NEFA: Non-esterified fatty acids
*n* − 3 PUFA: Omega-3 fatty acids
*n* − 6 PUFA: Omega-6 fatty acids
SFA: Saturated fatty acid
TC: Total cholesterol
TG: Triacylglycerol
TRL: Triacylglycerol-rich lipoprotein.
